# Radiolabelled FAPI Radiotracers in Oncology: A Comprehensive Review of Current Diagnostic and Emerging Therapeutic Applications

**DOI:** 10.3390/ph19010089

**Published:** 2026-01-02

**Authors:** Jolanta Czuczejko, Bogdan Małkowski, Jarosław Nuszkiewicz, Iga Hołyńska-Iwan, Paweł Waśniowski, Katarzyna Mądra-Gackowska, Wiktor Dróżdż, Karolina Szewczyk-Golec

**Affiliations:** 1 Nuclear Medicine Department, Oncology Centre-Prof. Franciszek Łukaszczyk Memorial Hospital, 2 Dr I. Romanowskiej St., 85-796 Bydgoszcz, Poland; pawel.wasniowski@cm.umk.pl; 2 Department of Psychiatry, Faculty of Medicine, Ludwik Rydygier Collegium Medicum in Bydgoszcz, Nicolaus Copernicus University in Toruń, 9 M. Curie Skłodowskiej St., 85-094 Bydgoszcz, Poland; wikdr@cm.umk.pl; 3 Department of Diagnostic Imaging, Faculty of Health Sciences, Ludwik Rydygier Collegium Medicum in Bydgoszcz, Nicolaus Copernicus University in Toruń, 2 Dr I. Romanowskiej St., 85-796 Bydgoszcz, Poland; malkowskib@co.bydgoszcz.pl; 4 Department of Medical Biology and Biochemistry, Faculty of Medicine, Ludwik Rydygier Collegium Medicum in Bydgoszcz, Nicolaus Copernicus University in Toruń, 24 Karłowicza St., 85-092 Bydgoszcz, Poland; jnuszkiewicz@cm.umk.pl; 5 Laboratory of Electrophysiology of Epithelial Tissue and Skin, Department of Pathobiochemistry and Clinical Chemistry, Faculty of Pharmacy, Ludwik Rydygier Collegium Medicum in Bydgoszcz, Nicolaus Copernicus University in Toruń, 9 M. Curie Skłodowskiej St., 85-094 Bydgoszcz, Poland; igaholynska@cm.umk.pl; 6 Department of Electroradiology, Collegium Medicum in Bydgoszcz, Nicolaus Copernicus University in Toruń, Sandomierska 16 St., 85-830 Bydgoszcz, Poland; 7 Department of Geriatrics, Faculty of Health Sciences, L. Rydygier Collegium Medicum in Bydgoszcz, Nicolaus Copernicus University in Toruń, Skłodowskiej Curie 9 St, 85-094 Bydgoszcz, Poland; katarzyna.madra@cm.umk.pl

**Keywords:** fibroblast activation protein (FAP), fibroblast activation protein inhibitor (FAPI), positron emission tomography (PET/CT), theranostics, cancer-associated fibroblasts, radionuclide therapy, molecular imaging

## Abstract

**Background/Objectives**: Fibroblast activation protein (FAP), which is abundantly expressed in cancer-associated fibroblasts (CAFs) across various epithelial malignancies, has emerged as a promising target for molecular imaging and radionuclide therapy. Although several reviews have addressed FAP-targeted diagnostics, a comprehensive synthesis integrating molecular biology, diagnostic performance, and early therapeutic development remains limited. This review summarises the current evidence on radionuclide-labelled FAP inhibitors (FAPIs), with particular emphasis on their diagnostic utility, emerging therapeutic applications, and the structural features that shape their biological behaviour. **Methods**: A structured literature search was conducted across PubMed, Scopus, and Web of Science, focusing on FAPI-based imaging and therapy. **Results**: Diagnostic studies consistently demonstrate high tumour-to-background contrast for [^68^Ga]Ga and [^18^F]-labelled FAPI radiotracers, particularly in tumours with prominent stromal components such as pancreatic, colorectal, breast, and head and neck cancers. FAPI PET/CT often surpasses [^18^F]FDG in lesion conspicuity in the brain, liver, and peritoneum. Therapeutic evidence shows encouraging tumour retention and safety profiles for agents such as [^177^Lu]Lu-FAP-2286 and [^90^Y]Y-FAPI-46, while α-emitting radiotracers (e.g., [^225^Ac]Ac-FAPI-04) demonstrate potent antitumor effects in preclinical models. **Conclusions**: Radiolabelled FAPI radiotracers hold significant potential as dual diagnostic and therapeutic agents, particularly for desmoplastic tumours with high CAF content. Nonetheless, clinical evidence remains in its early stages, and substantial questions persist regarding dosimetry, intertumoral variability in FAP expression, and optimal ligand selection for therapy. Continued development of next-generation FAPI constructs, along with well-designed prospective trials, will be crucial in defining the future role of FAPI-based theranostics in oncology.

## 1. Introduction

### 1.1. Biology and Function of CAFs

The tumour microenvironment (TME) refers to the complex ecosystem surrounding a tumour, composed of cancer cells, stromal cells, immune cells, the extracellular matrix (ECM), blood vessels, and various signalling molecules [1]. The TME plays a crucial role in tumour progression, metastasis, and resistance to therapy. Interactions between cancer cells and the TME are essential for cancer development.

The composition of stromal cells in solid tumours supports tumour progression, angiogenesis, and immune evasion. The main types of stromal cells are fibroblasts, endothelial cells of blood vessels, pericytes, and various immunocompetent cells [1,2,3]. While fibroblasts are not cancerous, their presence and activity within the tumour significantly impact its growth, progression, and metastasis.

The most prevalent stromal cell type consists of fibroblasts exhibiting the cancer-associated fibroblast (CAF) phenotype [1,4,5,6]. CAFs are characterised by a large, stellate shape, morphologically similar to smooth muscle cells, unlike resting fibroblasts, which have a spindle shape and a flattened, heterochromatic nucleus [4]. According to Xing et al. [4], CAFs likely originate from tissue-resident fibroblasts, epithelial cells, endothelial cells, or mesenchymal cells.

An active fibroblast phenotype, similar to that of CAFs, is observed in fibrotic lesions and during wound healing [7]. However, unlike fibroblasts involved in wound healing, which is a self-limiting process, tumour-associated fibroblasts remain persistently activated. A distinguishing feature of activated CAFs is their ability to synthesise and remodel the ECM in the desmoplastic stroma [7].

**Figure 1:**
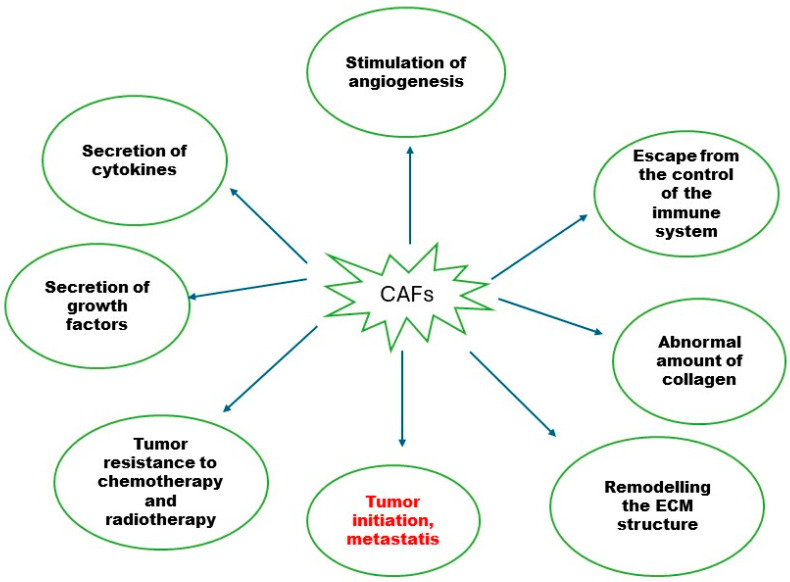
The role of cancer-associated fibroblasts (CAFs) in carcinogenesis. ECM—extracellular matrix.

CAFs are more metabolically active than untransformed fibroblasts [8]. As shown in Figure 1, CAFs play a crucial role in tumour progression by promoting the growth of invasive cells, facilitating the formation of new blood vessels, evading immune system surveillance, and enabling metastasis to distant organs [2,4,8]. CAFs contribute to cancer development through the secretion of cytokines (e.g., interleukin-6 (IL-6), C-X-C motif chemokine ligand 8 (CXCL8), C-X-C motif chemokine ligand 12 (CXCL12)), growth factors such as transforming growth factor-beta (TGF-β), hepatocyte growth factor (HGF), epidermal growth factor (EGF), fibroblast growth factor (FGF), and pro-angiogenic factors like vascular endothelial growth factor (VEGF) [8]. CAFs also produce excessive amounts of collagen, other extracellular matrix components, and acidic proteins [8].

Numerous studies have demonstrated the significant role of CAFs at various stages of carcinogenesis [9,10,11]. The direct stimulation of tumour cells by CAF-secreted compounds has been shown to enhance cell proliferation and promote the epithelial–mesenchymal transition (EMT), potentially transforming benign tumours into malignant ones [1,12]. Additionally, CAFs regulate tumour resistance to chemotherapy by releasing pro-tumoural factors (cytokines, chemokines, growth factors, etc.) or through direct adhesion to stromal fibroblasts and ECM components [8,13,14]. Moreover, CAFs are resistant to radiotherapy, and even irradiated fibroblasts can transform into a more activated CAF phenotype through paracrine interactions [8].

### 1.2. Fibroblast Activation Protein Overexpression and Cancerous Tumours

CAFs highly express fibroblast activation protein (FAP) in over 90% of human epithelial carcinomas [4,14,15,16,17,18]. According to a meta-analysis by Liu et al. [19], FAP overexpression has been identified in the microenvironment of 11 types of solid tumours, including colorectal cancer, pancreatic adenocarcinoma, non-small cell lung cancer, oesophageal cancer, gastric cancer, ovarian cancer, breast cancer, medullary thyroid cancer, endometrial cancer, squamous cell carcinoma, and bone sarcoma. Elevated FAP expression has been observed in fibroblasts forming the stroma of breast, colorectal, and pancreatic cancers [20]. In several cancer types, elevated FAP expression is associated with poor prognosis, underscoring its potential as a diagnostic and therapeutic target [2].

FAP, also known as seprase, is a type II transmembrane glycoprotein with both dipeptidyl peptidase and endopeptidase activities [2,3,4,6,15,21]. Notably, FAP is a cell surface protein found on tumour stromal fibroblasts rather than a secreted protease [15]. It belongs to the prolyl oligopeptidase family, characterised by a catalytic triad consisting of serine, aspartic acid, and histidine, and has a unique ability to cleave post-proline bonds [6]. Active FAP exists as a 170 kDa homodimer composed of two subunits, each weighing 97 kDa. Additionally, FAP can form a heteromeric active enzyme complex, exhibiting characteristics similar to dipeptidyl peptidase IV (DPP4) [6,14,15].

FAP expression is restricted to sites of tissue remodelling and activated stroma. It plays a role in the proteolysis of ECM components, facilitating cancer cell spread and metastasis, promoting tumour blood vessel formation, and supporting the nourishment of new cancer cells [6,15]. FAP’s substrates include neuropeptide Y, whose proteolytic product exhibits pro-angiogenic properties [6]. This underscores the critical role of FAP in the formation, remodelling, and maintenance of the TME [15].

In this context, García Megías et al. [22] recently published a comprehensive review of FAPI radiopharmaceuticals in nuclear oncology, integrating diagnostic and therapeutic applications within a conceptual framework rooted in tumour biology and the hallmarks of cancer. Building on this perspective, the present review offers a complementary synthesis of the available clinical evidence on the use of FAPI radiopharmaceuticals across solid tumours. While several recent reviews have comprehensively addressed the biological role of fibroblast activation protein and the clinical performance of FAPI-based radiopharmaceuticals in oncology, the present review aims to complement and extend these works by focusing on selected aspects that are less frequently discussed in the existing literature. In addition to summarising the diagnostic and theranostic applications of FAPI compounds, this article highlights emerging topics such as the biological and potential clinical relevance of soluble FAP (sFAP), practical considerations related to radionuclide production pathways (including generator- versus cyclotron-based approaches), and their implications for clinical translation and accessibility. By integrating biological, radiochemical, and logistical perspectives, this review provides a broader and practice-oriented synthesis of current evidence.

## 2. Results

### 2.1. FAP-Targeting Radiopharmaceuticals

Current knowledge about the role of fibroblasts in cancer development has provided the foundation for designing and applying multifunctional small-molecule compounds as carriers of radioactivity in tumours with FAP overexpression. These compounds can detect and treat neoplastic lesions through a crossfire effect [3,21,23]. Therapeutic radiotracers targeting FAP can degrade FAP and eliminate CAFs, potentially mitigating the negative impact of CAFs on chemotherapy and radiotherapy [14,24].

The absence of FAP in normal, healthy adult human tissues, with few exceptions, has led to the development of targeted therapies using radiolabelled FAP inhibitors (FAPIs) [6,17,25]. The large extracellular domain of FAP, with its catalytic site also located extracellularly, contributes to its suitability as a therapeutic target. This characteristic enables low background noise with high imaging contrast, minimises the likelihood of side effects, and allows for broad applicability across various tumour types. Additionally, it facilitates the design of combination therapies targeting both tumour cells and stromal components [2,3,26]. Targeting stromal cells has proven effective in combination with various therapeutic approaches, including antibodies, chimeric antigen receptor (CAR) T cells, immunoconjugates, peptide-drug complexes with consensus sequences for enzymatic activation, vaccines, and small molecules designed to inhibit FAP enzyme activity [14].

However, attention should be drawn to the findings of Lee et al. [27], who highlight their purification of a soluble, plasma-derived form of the fibroblast activation protein (sFAP). This derivative is generated either by cleavage at the Cys23-Ile24 bond within the transmembrane region or as an extracellular domain of FAP [26]. The authors [27] emphasise that its presence in plasma may influence the use of therapeutics designed to target FAP at the tissue level.

Among others, their discoveries are cited by the authors of a 2025 article [28], which aims to investigate the impact of various factors on the application of fibroblast activation protein inhibitors (FAPI)-targeting radiopharmaceuticals as a new approach to cancer treatment. Bilińska et al. [28] argue that sFAP in human plasma may significantly complicate the behaviour of FAPI radiopharmaceuticals in the body, affecting their absorption, biodistribution, and elimination. According to the authors [28], sFAP may compete with tumour-bound FAP, thereby influencing the specificity and pharmacokinetics of FAPI compounds. According to the authors [28], the potential interference of sFAP necessitates careful consideration of dosing strategies and may require adjustments to optimise the therapeutic efficacy and diagnostic accuracy of FAPI radiopharmaceuticals.

### 2.2. Development of FAP Inhibitors and Usefulness of Radiolabeled FAPI

In recent years, radiopharmacists have focused on labelling various small-molecule FAP inhibitors (FAPIs). Initially developed as conventional cancer therapeutics, FAP-specific inhibitors are now gaining importance as novel diagnostic tools for cancer treatment. For therapeutic applications, α- and β-emitting radionuclides such as [^177^Lu]lutetium, [^153^Sm]samarium, [^225^Ac]actinium, and [^90^Y]yttrium are being utilised [5,21,29,30]. In diagnostics, the most widely used positron-emitting isotopes include [^68^Ga]gallium, [^18^F]fluoride, and [^64^Cu]copper [3,5,21,24,26,31,32].

**Figure 2:**
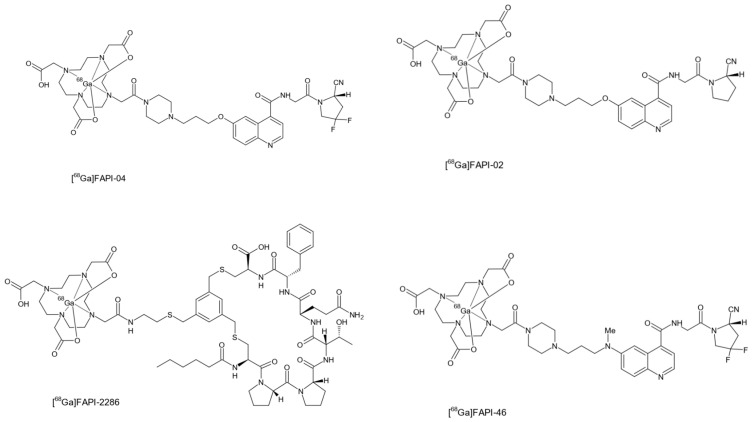
Chemical structure of FAPI-02, FAPI-04, FAPI-46, and FAPI-2286 with 1,4,7,10-Tetraazacyclododecane-1,4,7,10-tetraacetic acid (DOTA) chelator.

According to a meta-analysis conducted by Roustaei et al. [33], the ligands of interest include FAPI-02, FAPI-04, FAPI-05, FAPI-34, FAPI-46, FAPI-74, and FAPI-2286. Lindner et al. [24] and Dendl et al. [34] described the development of a series of FAPIs, including FA-PI-02, FAPI-04, FAPI-21, and FAPI-46, which were successfully labelled with [^68^Ga]gallium for imaging various tumours. Figure 2 illustrates the chemical structures of the most commonly used FAPI ligands, namely FAPI-02, FAPI-04, FAPI-46, and FAPI-2286, indicating the site where [^68^Ga]gallium is chelated.

Key pharmacokinetic differences between FAPI-04, FAPI-46 and FAPI-2286, including tumour retention and clearance pathways, are summarised in Table 1.

The typical structure of a small-molecule FAPI consists of chelator-linker-FAPI conjugates. The most widely used chelators include derivatives of DOTA (1,4,7,10-tetraazacyclododecane-N,N′,N″,N‴-tetraacetic acid), DATA (6-amino-1,4-diazepine-triacetic acid), and NOTA (1,4,7-triazacyclononane-1,4,7-triacetic acid) [30]. Common linkers include piperazine and squaramide [5,24]. Studies have demonstrated that the use of [^68^Ga]Ga-FAPI-02 and [^68^Ga]Ga-FAPI-04 in positron emission tomography (PET) diagnostics results in rapid renal clearance and good retention, with both radiotracers providing high-quality PET/computed tomography (CT) images and high tumour-to-background ratios [33,38]. FAPI-04, based on (4-quinolinyl)glycyl-2-cyanopyrrolidine, has shown the highest affinity for FAP [3,39].

According to Giesel et al. [35], [^68^Ga]Ga-FAPI-04 exhibits a longer residence time within lesions compared to FAPI-02 due to structural modifications in its proline ring (4,4-difluoroproline). When labelled with [^68^Ga]gallium, [^68^Ga]Ga-FAPI-04 has been found to possess the most favourable PET/CT imaging properties, including a nanomolar affinity for FAP, near-complete internalisation of FAP-related radioactivity, and rapid blood clearance [3]. [^68^Ga]Ga-DOTA-FAPI-04 PET/CT enables fast imaging with a clear tumour outline and high tumour-to-background contrast [39,40]. Additionally, its low background activity makes [^68^Ga]Ga-DOTA-FAPI-04 PET/CT particularly useful for assessing primary tumours in the brain, liver, and head and neck regions, as well as for detecting metastases in the liver and brain [41].

The use of radiolabelled [^68^Ga]Ga-FAPI-04 for PET/CT imaging in breast cancer patients was first demonstrated by Lindner et al. [24] in 2018. Recently, [^68^Ga]Ga-FAPI-04 PET/CT has been applied to the clinical detection of 28 different types of cancer [35,40]. However, according to Loktev et al. [36] and the meta-analysis by Roustaei et al. [33], [^68^Ga]Ga-FAPI-46 exhibits higher tumour uptake and lower uptake in normal organs compared to FAPI-04, making FAPI-46 a particularly promising ligand for diagnostic imaging and a theranostic agent [3,36].

Additionally, modifications in the FAPI-04 structure within the piperazine moiety (e.g., the methylene-bridged diaminobicycloheptane of FAPI-21) and the linker region (e.g., insertion of a methylamino group in FAPI-46) have resulted in significantly slower elimination of FAPI-21 and FAPI-46 from FAP-expressing cells [36]. The authors emphasise that the binding of FAPI to cells and its release rate from cells are influenced by multiple processes that have not yet been thoroughly studied [36].

### 2.3. Diagnostic Performance of [^68^Ga]Ga-FAPI vs. [^18^F]FDG

The effectiveness of fluorodeoxyglucose ([^18^F]FDG) and [^68^Ga]gallium/[^18^F]fluoride-FAPI derivatives has been the focus of numerous studies [21,23,35,38,39,40,41,42,43,44,45,46,47,48,49,50,51]. A comparison of [^68^Ga]FAPI versus [^18^F]FDG uptake in specific types of cancer, based on selected articles, is presented in Table 2.

[^68^Ga]Ga-FAPI has also been used as an adjunct to [^18^F]FDG diagnostics to achieve more precise tumour localisation for planned surgery or radiotherapy [2,45]. Currently, [^18^F]FDG remains the most frequently used radiopharmaceutical for PET/CT and PET/magnetic resonance imaging (MRI) diagnostics, as well as for recurrence assessment, therapy planning, and evaluation [25].

However, [^18^F]FDG has certain limitations, such as a lack of specificity, high physiologic uptake in the brain, liver, urinary tract, and inflamed areas, as well as limited sensitivity in detecting peritoneal carcinomatosis [17,35,38,44]. Additionally, [^18^F]FDG is not suitable for detecting certain types of cancer, including mucin-secreting epithelial malignancies, lepidic adenocarcinomas, well-differentiated neuroendocrine tumours, and well-differentiated endocrine gland malignancies [46]. Chen et al. [41,51] highlight the significance of [^68^Ga]Ga-DOTA-FAPI-04 in diagnosing various types of primary cancer and metastatic lesions, particularly pancreatic, gastric, liver, and skeletal cancers, as well as lymph node metastases. The poor diagnostic performance of [^18^F]FDG affects tissues with an increased physiological demand for glucose.

### 2.4. Application of Radiolabeled FAPI and [^18^F]FDG in Multi-Cancer Studies

In a study by Ballal et al. [23], patients with breast, lung, head and neck, gall bladder, and ovary cancers were diagnosed using both [^18^F]FDG and [^68^Ga]Ga-DOTA.SA.FAPi. PET/CT scans were performed 60 min after administering the radiopharmaceuticals—a rapid but stable uptake of [^68^Ga]Ga-DOTA.SA.FAPi was observed in malignant lesions within 10 min of intravenous injection. Additionally, the radiotracer demonstrated rapid clearance from non-target organs, thereby reducing radiation exposure to normal tissues.

The physiological uptake of [^68^Ga]Ga-DOTA.SA.FAPi and [^18^F]FDG in the liver, kidneys, heart tissue, lacrimal glands, oral mucosa, salivary glands, and thyroid gland were similar, except in the pancreas, where the uptake of [^68^Ga]Ga-DOTA.SA.FAPi was significantly higher than that of [^18^F]FDG. In contrast to the substantial uptake of [^18^F]FDG in normal brain parenchyma, negligible uptake was noted with [^68^Ga]Ga-FAPI, suggesting that [^68^Ga]Ga-FAPI may be a valuable marker for detecting brain metastases.

Ballal et al. [23] demonstrated that [^68^Ga]Ga-DOTA.SA.FAPi and [^18^F]FDG had similar efficacy in detecting breast, head, and neck cancers, with 94.4% of patients showing consistent lung metastasis detection between the two radiotracers. The authors concluded that the diagnostic accuracy of [^68^Ga]Ga-DOTA.SA.FAPi PET/CT is comparable to standard [^18^F]FDG-PET/CT imaging.

Giesel et al. [35] compared [^68^Ga]Ga-FAPI PET/CT to [^18^F]FDG PET/CT in 71 patients with various types of cancer (e.g., head and neck cancer, lung carcinoma, biliary-pancreatic cancer, gastrointestinal tract cancer, gynaecological cancer, neuroendocrine bladder carcinoma, prostate carcinoma, B-cell lymphoma, synovial sarcoma of the lung, adrenal gland carcinoma, malignant solitary fibrous tumour, and cancers of unknown primary origin). The analysis revealed that [^68^Ga]Ga-FAPI uptake was very low in most healthy tissues compared to [^18^F]FDG uptake, and the tumour-to-background ratios for [^68^Ga]Ga-FAPI were comparable to or superior to those of [^18^F]FDG. However, no significant differences in the mean maximum standardised uptake value (SUVmax) were found between [^68^Ga]Ga-FAPI and [^18^F]FDG in primary or metastatic lesions overall, except in two individual cases (one with ovarian cancer and another with pancreatic cancer), where notable differences were observed.

Results obtained by Chen et al. [41] indicated that PET/CT with [^68^Ga]Ga-DOTA-FAPI-04 was able to detect almost all primary lesions (55 out of 56), whereas [^18^F]FDG missed 10 of them (including gastric, pancreatic, hepatocellular, and cervical cancers, lung adenocarcinoma, cholangiocarcinoma, and diffuse astrocytoma). The sensitivity of [^18^F]FDG for imaging primary lesions was lower (82.1%) compared to [^68^Ga]Ga-DOTA-FAPI-04 (98.2%). For metastatic lesions, [^68^Ga]Ga-DOTA-FAPI-04 demonstrated significantly higher uptake than [^18^F]FDG [35]. Additionally, [^68^Ga]Ga-DOTA-FAPI-04 PET/CT identified more positive lymph nodes than [^18^F]FDG PET/CT in the neck, supraclavicular, mediastinal, and abdominal regions. Thus, [^68^Ga]Ga-DOTA-FAPI-04 was more sensitive and accurate, though not necessarily more specific [41]. Conversely, Civan et al. [50] found that lymph node metastases exhibited higher SUVmax values on [^18^F]FDG PET/CT.

Another study by Chen et al. [51] reported that in 59 patients, [^18^F]FDG PET/CT yielded negative or unclear results for primary tumours in 22 cases. At the same time, [^68^Ga]Ga-DOTA-FAPI-04 PET/CT showed high tracer uptake in most primary lesions (86.4%), particularly in liver and gastric cancers. Additionally, [^68^Ga]Ga-DOTA-FAPI-04 PET/CT was superior in identifying metastatic lesions, especially in peritoneal carcinomatosis, liver metastases, and skeletal metastases [51]. The authors highlighted the limitations of [^18^F]FDG in diagnosing gastric cancer, due to histological variations that affect [^18^F]FDG uptake [51]. In uncertain lesions where [^18^F]FDG PET/CT is less effective, [^68^Ga]Ga-DOTA-FAPI-04 appears to be a valuable alternative.

Compared to [^18^F]FDG, [^68^Ga]Ga-DOTA-FAPI-04 PET/CT images demonstrated higher tumour-to-background contrast in liver cancer, nasopharyngeal cancer, and glioma [41], as well as in brain metastases [51]. In five cases, [^68^Ga]Ga-DOTA-FAPI-04 showed better delineation of brain metastases than [^18^F]FDG PET/CT [51].

Chen et al. [41] also highlighted the diagnostic value of [^68^Ga]Ga-DOTA-FAPI-04 in detecting small neoplastic lesions. Tiny tumours (diameter < 1.0 cm) were identified using [^68^Ga]Ga-DOTA-FAPI-04 but were not visible on [^18^F]FDG PET/CT. This finding was supported by Wang et al. [49], who concluded that [^68^Ga]Ga-FAPI-04 PET/CT is more sensitive than [^18^F]FDG PET/CT in detecting small, well-differentiated, or moderately differentiated intrahepatic neoplastic lesions.

Despite its benefits, researchers have identified weaknesses in using FAPI as a diagnostic radiopharmaceutical [41,51]. For example, Chen et al. [41] observed that [^68^Ga]Ga-DOTA-FAPI-04 was not more tumour-specific than [^18^F]FDG in a case of pancreatic cancer, as intense uptake in the entire pancreas masked the presence of the primary lesion. Additionally, false-positive findings related to IgG4-related disease have been reported, highlighting the need for caution when using [^68^Ga]Ga-DOTA-FAPI-04 in cancer diagnosis, particularly in cases associated with inflammation [41]. False-positive results have also been observed in the diagnosis of diseases characterised by pathological fibrosis, such as myelofibrosis, granulomatous diseases, and cirrhosis [41].

A retrospective study of 56 adult patients with histopathologically confirmed colorectal cancer compared [^68^Ga]Ga-FAPI-04 PET/CT with [^18^F]FDG PET/CT [52]. Among the patients diagnosed with colon cancer, 20% had metastatic lesions, and 40% experienced tumour relapse after prior treatment for localised disease. Based on CT, MRI, colonoscopy, or surgical pathology results at one year, 14 patients experienced primary tumour recurrence, 9 had lymph node metastases, and 5 had ovarian metastases. [^68^Ga]Ga-FAPI-04 PET/CT demonstrated higher specificity and sensitivity in imaging primary tumours and was more sensitive than [^18^F]FDG PET/CT in detecting lymph node metastases, particularly peritoneal metastases and bowel implants. However, the sensitivity of [^68^Ga]Ga-FAPI-04 PET/CT for bone metastases appeared lower than that of [^18^F]FDG PET/CT. Overall, the study demonstrated that [^68^Ga]Ga-FAPI-04 PET/CT provided more precise visualisation of lesions than [^18^F]FDG PET/CT.

### 2.5. The Impact of FAP Imaging on Lung Cancer

In the study by Xi et al. [52], low uptake of both [^18^F]FDG PET/CT and [^68^Ga]Ga-FAPI-04 PET/CT was observed in lung metastatic lesions in five patients with colorectal carcinoma.

In a study conducted by Chen et al. [53] involving 73 patients with lung cancer, the authors demonstrated that FAP expression levels depend on the cancer subtype. Specifically, in non-small cell lung cancer (NSCLC), FAP levels increased by 100%. In contrast, in small cell lung cancer (SCLC) and large cell neuroendocrine carcinoma (LCNC), FAP expression increased by 67%.

Wei et al. [54] studied 61 patients with lung cancer and demonstrated a correlation between histochemically determined FAP expression in lung cancer and [^18^F]AlF-NOTA-FAPI-04 uptake in PET/CT. The authors found that uptake values of [^18^F]AlF-NOTA-FAPI-04 in metastases were highest in squamous cell carcinoma, followed by adenocarcinoma, with the lowest uptake observed in metastases from SCLC. However, no significant differences in uptake values were observed among primary tumours of different pathological lung cancer subtypes. The authors emphasised that PET/CT imaging with [^18^F]AlF-NOTA-FAPI-04 may be valuable for therapy planning in patients with advanced lung cancer.

Wang et al. [48] conducted a study on six patients, performing dynamic whole-body PET/CT examinations using [^68^Ga]Ga-FAPI-04, followed by a static [^18^F]FDG scan within a week after the initial imaging. The study began with a low-dose CT scan (120 kV, 35 mA) followed by the injection of [^68^Ga]Ga-FAPI-04. Subsequently, a whole-body dynamic PET scan was performed immediately after intravenous injection of [^68^Ga]Ga-FAPI-04 (at activity levels ranging from 103.2 to 257.5 MBq) and continued for six frames, up to 60 min post-injection. All subjects underwent a follow-up static [^18^F]FDG PET/CT scan within a week of the [^68^Ga]Ga-FAPI-04 imaging. The study revealed that [^68^Ga]Ga-FAPI-04 was rapidly cleared from the body through renal excretion, resulting in a low background signal. The authors [48] demonstrated that the variability of [^68^Ga]Ga-FAPI-04 uptake in normal lung tissue was lower than that of [^18^F]FDG. Moreover, high-quality lung cancer diagnostic images could be obtained more quickly with [^68^Ga]Ga-FAPI-04 than with [^18^F]FDG.

### 2.6. Diagnostic Value of [^68^Ga]Ga-FAPI-PET/CT vs. [^18^F]FDG -PET in Gynaecological Tumours and Breast Cancer

Dendl et al. [55] investigated [^68^Ga]Ga-FAPI-PET/CT as a radiopharmaceutical for complementary diagnostics, with potential superiority over [^18^F]FDG-PET in imaging gynaecological tumours. The study involved two patient groups. The first group consisted of 31 patients with various gynaecological cancers (including breast, ovarian, cervical, endometrial, and fallopian tube cancers, as well as uterine leiomyosarcoma) and included both primary and metastatic cases. The second group comprised 167 patients with various malignancies (head and neck cancer, lung cancer, pancreatic cancer, gastrointestinal cancer, and others) to analyse [^68^Ga]Ga-FAPI uptake in hormone-responsive organs such as the endometrium, ovaries, and breasts. Notably, the study revealed a low uptake of the radiopharmaceutical in healthy breast tissue but a relatively high mean SUVmax in breast cancer. Additionally, FAPI uptake by histological classification was significantly higher in high-grade lesions than in low-grade ones. The authors also found that hormone-responsive organs were associated with increased FAPI uptake in PET/CT.

In a study by Xi [44], [^68^Ga]Ga-FAPI-04 PET/MR and [^18^F]FDG PET/CT demonstrated complementary advantages. While [^18^F]FDG-PET/CT was more effective in assessing the degree of tumour malignancy, [^68^Ga]Ga-FAPI-04 PET/MR was more valuable in predicting incomplete resectability in ovarian cancer cases. Although [^68^Ga]Ga-FAPI-04 PET/MR was less effective than [^18^F]FDG in diagnosing primary tumours, it was significantly more valuable in detecting peri-oesophageal and peritoneal metastases, as well as metastases in the digestive tract.

In turn, Sahin et al. [56] investigated whether [^68^Ga]Ga-FAPI PET/CT could be a better alternative to the standard [^18^F]FDG PET/CT for staging Invasive Lobular Carcinoma (ILC) of the breast. In this study, 23 female patients (mean age 51) with hormone-positive, HER2-negative ILC underwent both scans within a week, without intervening treatment. Lesions were visually assessed and quantified using SUVmax, tumour-to-background, and uptake ratios. Authors [56] demonstrated that [^68^Ga]Ga-FAPI PET/CT significantly outperformed [^18^F]-FDG PET/CT. Higher tumoral activity and tumour-to-background uptake ratios were observed, resulting in increased uptake in primary tumours. This led to the detection of additional foci, including multicentric cancer, and more frequent detection of axillary lymph node metastases with higher uptake values. Additionally, a greater number of lesions were identified overall, including bone and liver metastases. In this study, [^68^Ga]Ga-FAPI PET/CT demonstrated superior sensitivity to [^18^F]-FDG PET/CT for detecting primary tumours, lymph node involvement, and distant metastases in ILC patients. This suggests it is a promising alternative imaging modality for staging this specific breast cancer subtype.

### 2.7. PET/CT Imaging with [^68^Ga]Ga-FAPI and [^18^F]FDG in Glioma

Research by Röhrich et al. [57] revealed that PET/CT imaging with [^68^Ga]Ga-FAPI is a promising and independent method for visualising gliomas, providing complementary information to MRI. Although the biological basis of FAP-specific signalling in gliomas is not fully understood, given the absence of reactive fibroblasts in the brain, the findings suggest that [^68^Ga]Ga-FAPI holds potential for clinical application. However, further in-depth research is needed to explore the mechanisms and clinical utility of FAP radiopharmaceuticals in gliomas. Notably, PET/CT imaging with [^68^Ga]Ga-FAPI may be valuable for biopsy planning and distinguishing between pseudoprogression and actual tumour progression after radiotherapy.

### 2.8. The Role of [^68^Ga]Ga-FAPI PET/CT in Renal Cell Carcinoma

In a preliminary study, Civan et al. [50] investigated the role of [^68^Ga]Ga-FAPI-04 in PET/CT diagnostics and the uptake patterns of primary and metastatic lesions in patients with renal cell carcinoma (RCC). The authors emphasised that this was the first study to explore the utility of this radiopharmaceutical in diagnosing RCC.

In an earlier study, Kratochwil et al. [40] examined a single case of RCC and observed very low [^68^Ga]Ga-FAPI-04 uptake (SUVmax < 6).

The study by Civan et al. [50] involved 20 patients, including those with highly suspicious renal masses, newly diagnosed RCC patients with suspected distant metastases for staging, and patients undergoing restaging for known or suspected RCC metastases. The findings revealed that 49% of all lesions demonstrated moderate to high FAPI uptake with SUVmax ≥ 6, most of which were primary or recurrent tumours and bone metastases.

Additionally, all recurrent lesions showed higher tumour-to-background ratios on [^68^Ga]Ga-FAPI-04 PET/CT compared to [^18^F]FDG-PET. Significantly higher SUVmax values were noted in lung metastases, and higher SUVmax and tumour-to-background ratios in bone metastases.

However, the authors [50] also observed substantial discrepancies in the PET/CT SUV values of [^68^Ga]Ga-FAPI-04 among patients, which could be attributed to the heterogeneous nature of RCC. Due to the small cohort size, they concluded that FAPI may have a prognostic and complementary role in the diagnosis of RCC’s PET/CT, warranting further investigation in more extensive studies [50].

### 2.9. [^68^Ga]Ga-FAPI-04 PET/CT as a Diagnostic Biomarker in Well-Differentiated Neuroendocrine Tumours

Neuroendocrine neoplasms (NENs) are a rare, heterogeneous group of tumours characterised by variations in clinical presentation, developmental biology, and morphological appearance. NENs originate from specialised neuroendocrine cells distributed throughout the body, particularly in the epithelium of the gastric mucosa, intestinal villi and crypts, bile ducts, pancreatic islets, lungs, thymus, skin, prostate, adrenal glands, thyroid, kidneys, hypothalamus, pituitary, and paraganglionic bodies of the parasympathetic and sympathetic nervous systems [58].

NENs are classified into three groups: neuroendocrine tumours (NETs), neuroendocrine carcinomas (NECs), and mixed neuroendocrine neoplasms [58]. Approximately 70% of all NETs are gastroenteropancreatic neuroendocrine tumours (GEP-NETs). A key characteristic of NEN cells is the presence of surface somatostatin receptors (SSTRs), which can be effectively imaged using [^68^Ga]Ga-DOTA-peptides, such as [^68^Ga]Ga-DOTANOC, [^68^Ga]Ga-DOTATOC, and [^68^Ga]Ga-DOTATATE [59].

Simsek et al. [60] conducted a prospective study comparing the use of [^68^Ga]Ga-DOTATATE PET/CT and [^68^Ga]Ga-FAPI-04 PET/CT as diagnostic biomarkers in well-differentiated NETs. Twelve patients with metastatic NETs who had received at least two cycles of [^177^Lu]Lu-DOTATATE therapy and exhibited disease progression were included. The study concluded that the median SUVmax levels and the number of detected tumours were significantly higher in [^68^Ga]Ga-DOTATATE PET/CT (*p* < 0.001). In only one patient, tumour uptake was higher in [^68^Ga]Ga-FAPI-04 PET/CT compared to [^68^Ga]Ga-DOTATATE PET/CT. Simsek et al. [60] concluded that [^68^Ga]Ga-FAPI-04 PET/CT was largely ineffective in well-differentiated NETs refractory to [^177^Lu]Lu-DOTATATE therapy, indicating a limited diagnostic role for this imaging modality.

Moreover, [^68^Ga]Ga-FAPI-46 proved to be ineffective in diagnosing extrahepatic NETs (EBNETs), whereas [^68^Ga]Ga-DOTATATE PET/CT demonstrated increased tracer uptake at the affected site [61]. The most common sites of NETs include the gastrointestinal tract (67.5%) and the bronchopulmonary system (25.3%), with only 0.1–0.4% occurring in the extrahepatic bile ducts. Given that [^68^Ga] Ga-FAPI PET/CT targets cancer-associated FAP, which is linked to an intense desmoplastic reaction, the authors suggest that extrahepatic NETs do not exhibit significant desmoplastic activity, explaining the poor efficacy of [^68^Ga]Ga-FAPI-04 in detecting these tumours.

In addition to the extent of desmoplastic reaction, tumour grade may represent an important factor influencing FAPI uptake in neuroendocrine neoplasms. Well-differentiated NETs (G1–G2) are typically characterised by a preserved stromal architecture, low fibroblast activation, and high somatostatin receptor expression, which collectively may explain the limited diagnostic performance of FAPI-based imaging in this subgroup [60,61]. In contrast, high-grade or dedifferentiated neuroendocrine carcinomas often exhibit increased tumour aggressiveness, stromal remodelling, and loss of somatostatin receptor expression, raising the possibility that FAPI PET/CT could provide complementary information in these cases [58,59]. Although direct evidence correlating FAP expression with NET grade remains limited, this hypothesis is biologically plausible and warrants further investigation, particularly in patients with SSTR-negative or therapy-refractory neuroendocrine tumours (NETs) [25,58,59,60,61].

### 2.10. Advantages and Disadvantages of [^18^F]FDG and [^68^Ga]Ga-FAPI Due to the Method of Production and Use in Patients

Both the synthesis methods and the requirements for administering [^18^F]FDG and [^68^Ga]Ga-FAPI to patients have their respective advantages and disadvantages. Administering [^18^F]FDG requires specific patient preparation, which can involve several inconveniences [38]. These include prolonged radiation exposure due to the approximately 60 min waiting period between tracer administration and examination. Additionally, [^18^F]FDG has limitations in imaging neoplastic lesions in tissues with high glucose metabolism and necessitates patient fasting [38]. This can be particularly burdensome for diabetic patients, who must abstain from eating for approximately six hours while managing their diabetes medications [62].

In contrast, [^68^Ga] radiolabelled FAPI administration does not require fasting, and there are no contraindications for diabetic patients. Another advantage of [^68^Ga]Ga-FAPI is its ability to initiate the scan just minutes after administration, thereby reducing patient radiation exposure and enhancing workflow efficiency for medical staff [62].

A significant advantage of using FAPI derivatives labelled with [^68^Ga]gallium is the ease of obtaining the positron emitter from a ^68^Ge/^68^Ga generator. The elution process for [^68^Ga]gallium is quick and straightforward, allowing any nuclear medicine laboratory equipped with a ^68^Ge/^68^Ga generator to prepare gallium-labelled FAPI derivatives on-site. In contrast, producing [^18^F]fluoride requires a cyclotron and a manufacturing permit, making it more complex and costly [63].

However, there are limitations to using [^68^Ga]Ga-FAPI. The eluate obtained from the ^68^Ge/^68^Ga generator has relatively low activity compared to the higher activity of [^18^F]fluoride produced by a cyclotron. The typical maximal batch size obtained from a ^68^Ge/^68^Ga generator is 2–4 GBq, which decreases due to the half-life of ^68^Ge (271 days) [64]. Additionally, [^68^Ga]gallium has a shorter half-life (68 min) compared to [^18^F]fluoride (110 min). As a result, the currently used methods with [^68^Ga]Ga-FAPI are not well-suited for testing large numbers of patients and require an on-site and on-time synthesis of the radiotracer [64]. In contrast, the production of [^18^F]FDG enables the preparation of significantly more doses per batch compared to a few doses of [^68^Ga]Ga-FAPI, which is crucial for large hospital centres [65].

Beyond production logistics, physical properties of the radionuclide also play a crucial role in image quality. In particular, [^18^F] is characterised by a lower positron energy and shorter positron range compared to [^68^Ga], which generally translates into improved spatial resolution and sharper lesion delineation in PET imaging [46,63]. These physical advantages may be particularly relevant for detecting small lesions or those located in anatomically complex regions. Consequently, [^18^F]-labelled FAPI radiopharmaceuticals may offer superior image quality compared to their [^68^Ga]-labelled counterparts, complementing the logistical benefits associated with cyclotron-based production [65,66].

To address this issue, clinical trials are exploring the use of various FAPI molecules labelled with [^18^F]fluoride. Findings by Yang et al. [43] suggest that [^18^F]-FAPI-04 exhibited higher tracer uptake and outperformed [^18^F]-FDG PET/CT in detecting primary and metastatic lesions in patients with gastrointestinal system cancers. In turn, Giesel et al. [65] and Watabe et al. [66] evaluated the effectiveness of FAPI-74 labelled with [^18^F]fluoride in patients with lung cancer as well as various histopathologically confirmed cancers and benign lesions.

The authors [65,66] reported that the FAPI-74 ligand is well-suited for labelling with [^18^F]fluoride, allowing for the large-scale batch production of [^18^F]F-FAPI-74. The biodistribution and optimal tumour-to-background ratios of [^18^F]F-FAPI-74 were comparable to those of [^68^Ga] gallium-labelled FAPI. Research by Watabe et al. [66] revealed significantly higher uptake of [^18^F]F-FAPI-74 in primary and metastatic lesions compared to [^18^F]FDG PET, indicating that [^18^F]F-FAPI-74 could be a promising diagnostic marker. However, the sample sizes in these studies remain limited, underscoring the need for further investigation.

An exciting aspect of the research is the use of the copper-64 (^64^Cu) radioisotope as a positron-emitting isotope in PET imaging [31,32]. ^64^Cu emits positrons (β^+^, 17.8%) with an energy of 0.655 MeV, providing high-quality PET imaging. Additionally, copper-64 emits beta-minus radiation (β^−^, 38.4%), and due to this β^−^ emission, ^64^Cu can be used not only for diagnostics but also for targeted radiotherapy, particularly in combination with ^67^Cu, which is purely therapeutic [31,67]. The half-life of ^64^Cu—12.7 h is longer than that of commonly used ^68^Ga and ^18^F [31], which, in my opinion, allows for more flexible imaging schedules and transportation to distant facilities.

### 2.11. Theranostic Use of Radiolabeled FAPI

Therapeutic applications of radiolabelled FAPI ligands are still in an early stage of development, yet several clinically relevant patterns are emerging. The therapeutic potential of radiolabelled FAPI has been explored in numerous preclinical and clinical studies, involving various types of FAP ligands, including FAPI-02, FAPI-04, FAPI-13, FAPI-46, FAP-2286, and SA.FAPI, ND-bisFAPI, PNT6555, and FAP radiotracers with albumin-binding moieties have been the focus of these studies [68]. Researchers are not only exploring different FAPI ligands but also focusing on selecting appropriate therapeutic isotopes, such as [^177^Lu]lutetium, [^225^Ac]actinium, and [^90^Y]yttrium, which have shown significant potential.

Among these isotopes, [^177^Lu]lutetium emits medium-energy β^−^ particles and gamma rays visible in imaging tests, with a half-life (t_1/2_) of 6.647 days. Its low-energy emissions make it ideal for targeting and destroying small clusters of cancer cells. On the other hand, [^225^Ac]actinium is an alpha particle emitter with a t_1/2_ of 10 days, making it suitable for treating small tumours due to its high linear energy transfer and short tissue penetration range. [^225^Ac]actinium delivers high, precise energy to tumours per unit of radioactivity, leading to double-strand DNA breaks. In contrast, [^90^Y]yttrium (t_1/2_ = 64 h) emits high-energy β^−^ particles, which are effective in treating large deposits of cancer cells [69,70].

Lindner et al. [24] reported studies on labelling FAPI-02 and FAPI-04 with [^177^Lu]lutetium. The authors successfully developed FAPI-04 as a theranostic tool in pre-clinical studies, demonstrating faster internalisation of [^177^Lu]Lu-FAPI-04 in FAP-positive tumours compared to [^177^Lu]Lu-FAPI-02, as well as a shorter blood retention time compared to [^177^Lu]Lu-FAPI-13.

Baum et al. [37] investigated the use of [^68^Ga]Ga-FAP-2286 and [^68^Ga]Ga-FAPI-04 for diagnostics and [^177^Lu]Lu-FAP-2286 for therapy in 11 patients with progressive and metastatic adenocarcinomas of the pancreas, breast, ovary, and rectum. The patients received an average activity of 5.8 ± 2.0 GBq (2.4–9.9 GBq). Post-treatment single-photon emission computed tomography (SPECT)/CT scans revealed excellent uptake of [^177^Lu]Lu-FAP-2286 in cancerous lesions (from 72 h to 10 days post-administration) in all patients. No adverse pharmacological effects were observed, and two patients reported significant pain relief. The authors stated that [37], unlike smaller FAPI molecules such as FAPI-02 and FA-PI-04, [^177^Lu]Lu-FAP-2286 exhibited prolonged tumour retention (effective tumour half-life: mean of 44 h for bone metastases and 32 h for single liver metastases), indicating its more significant therapeutic potential.

Following Baum et al. [37], it was suggested that using lower doses of [^90^Y]Y-FAPI-2286 compared to [^177^Lu]Lu-FAP-2286 could help reduce cancer progression due to the higher energy emitted by [^90^Y]yttrium β^−^ particles. The concept of using an alpha emitter, [^225^Ac]actinium, was explored by Watabe et al. [31]. In their preclinical research, dynamic PET scans with [^64^Cu]Cu-FAPI-04 and treatment with 34 KBq of [^225^Ac]Ac-FAPI-04 per mouse demonstrated that the combination of [^64^Cu]Cu-FAPI-04 and [^225^Ac]Ac-FAPI-04 could be effective for theranostic treatment of FAP-expressing pancreatic cancer. The authors [31] observed tumour shrinkage to 1.54 ± 0.65 cm^3^ after approximately 20 days in mice treated with ^[225^Ac]Ac-FAPI-04.

Ballal et al. [5] developed FAPI radiotracers with squaramide subunits linked to bifunctional DOTA/DATA5m chelators and FAP inhibitors as targeting moieties. These molecules demonstrated promising theranostic properties in vitro and in preclinical and clinical studies across various cancer types [23,42].

Gallium-68-labelled FAPI ligands, such as FAPI-04 and FAPI-46, exhibit high tumour-to-background ratios (e.g., TBR > 6 in pancreatic and breast cancer lesions), allowing for the sensitive detection of FAP-positive tumours. In therapeutic applications, beta–emitter–labelled compounds such as [^177^Lu]Lu-FAP-2286 or [^90^Y]Y-FAPI-46 have shown encouraging results in early clinical studies, including partial responses or disease stabilisation in a subset of patients, with mostly manageable hematologic toxicity. However, evidence remains limited to small, uncontrolled cohorts, and longer-term outcomes are not yet established [21,23].

Unfortunately, monomeric [^177^Lu]Lu-DOTA.SA.FAPI was found to be eliminated from the body after 48 h. To address this, Moon et al. [71] developed dimeric forms (DO-TA(SA.FAPi)_2_ and DOTAGA.(SA.FAPi)_2_) that exhibited longer tumour retention, faster internalisation, higher affinity, and quicker clearance from non-target organs, presenting a novel approach for treating various cancers using a theranostic method.

In 2018, Lindner et al. [24] treated a patient with metastatic breast cancer using 2.9 GBq of [^90^Y]Y-FAPI-04. The researchers reported significant [^90^Y]Y-FAPI-04 accumulation three hours post-administration, leading to pain reduction and a decreased morphine dose.

In another study, Ferdinandus et al. [30] investigated [^90^Y]Y-FAPI-46 therapy in patients with solid tumours, including soft tissue or bone sarcomas and pancreatic cancer. All patients had previously undergone conventional treatments or were ineligible for other therapies. Before treatment, patients underwent PET imaging with [^68^Ga]Ga-FAPI-46, which showed that more than 50% of lesions had a positive result (SUVmax ≥ 10). Patients received an average dose of 3.8 GBq of [^90^Y]Y-FAPI-46 in the first cycle and 7.4 GBq in the second. The study concluded that [^90^Y]Y-FAPI-46 was well-tolerated, with a low incidence of adverse events, including thrombocytopenia. Although therapeutic efficacy was observed, further trials with larger patient cohorts are needed to confirm the treatment’s effectiveness and toxicity profile.

Clinical trials are underway to evaluate the diagnostic and therapeutic efficacy of radiolabelled FAP-2286 derivatives. One such multi-arm, prospective Phase 1 trial is assessing novel imaging agents for detecting metastatic cancer in patients with solid tumours using [^68^Ga]gallium or [^64^Cu]copper FAP-2286 radiotracers [72]. This study aims to image breast, pancreatic, sarcoma, prostate, bladder, colon, and head and neck cancers, evaluating radiotracer uptake and retention in various solid tumours, the ability to detect metastatic disease, and dosimetry.

Another clinical trial is focused on assessing the safety and efficacy of [^177^Lu]Lu-FAP-2286 as monotherapy (Phase 2) in participants with pancreatic ductal adenocarcinoma (PDAC), non-small cell lung cancer (NSCLC, and breast cancer, as well as in combination with chemotherapy for untreated PDAC or relapsed NSCLC [73]. In this trial, up to six doses of [^177^Lu]Lu-FAP-2286 will be administered every six weeks in Phase 1 and every four weeks in Phase 2. All participants will be monitored for safety throughout treatment and assessed for disease status.

## 3. Materials and Methods

A structured literature search was conducted in PubMed, Scopus, and Web of Science. The search terms included “FAPI,” “fibroblast activation protein inhibitor,” “FAP-targeted imaging,” “FAP-targeted therapy,” “radiotheranostics,” “FAP-2286,” and “FAPI PET.” Only original research articles, clinical studies, and preclinical investigations involving radio-labelled FAPI ligands were included. Reviews, non-radiolabelled FAP-targeting strategies, and studies with insufficient methodological detail were excluded. Although this review was not registered as a systematic review, PRISMA principles were followed for study identification and selection to enhance transparency and reproducibility.

## 4. Discussion and Conclusions

Recent comprehensive reviews, including that by García Megías et al. [22], have positioned FAPI radiopharmaceuticals within a broader biological and theranostic framework, emphasising the central role of fibroblast activation protein in shaping the tumour microenvironment and multiple hallmarks of cancer. While confirming the high diagnostic performance of FAPI PET/CT across a wide range of solid tumours, these authors also highlight the heterogeneity of FAP expression and the resulting variability in radiopharmaceutical uptake among different tumour entities. Importantly, FAPI-based imaging is not proposed as a universal replacement for [^18^F]FDG, but rather as a complementary modality with particular advantages in desmoplastic and stroma-rich malignancies.

Earlier reviews [2,14,25,33,34,43,68] have primarily focused on the molecular characteristics of FAPI ligands, radiochemistry, biodistribution profiles, and initial clinical imaging performance of selected compounds, often in limited patient cohorts or specific tumour types. In contrast, the present review provides a structured synthesis of available clinical evidence across a broad spectrum of solid tumours, highlighting tumour-specific uptake patterns, inter-study variability, and current limitations relevant for clinical translation, particularly in the context of emerging FAPI-targeted radionuclide therapy. Together, these observations support the role of FAPI-based imaging and theranostics as a rapidly evolving but still developing field, in which further standardisation and prospective studies are required to define their precise clinical utility.

Primarily, the present manuscript provides added value by synthesising several aspects that have not yet been jointly addressed. Specifically, this review:

1. Integrates newly emerging data on soluble FAP (sFAP) and its implications for FAPI biodistribution and therapeutic efficacy.
2. Compares the performance of both gallium-68 and fluorine-18 FAPI radiotracers in relation to generator-based vs. cyclotron-based production constraints.
3. Summarises early therapeutic studies involving multimeric and albumin-binding FAPI constructs; and
4. Discusses current challenges associated with heterogeneous stromal composition and fibroblast-related tumour biology.

By combining molecular mechanisms, diagnostic applications, and evolving therapeutic strategies, this review aims to bridge the gap between preclinical findings and the emerging clinical landscape, with a particular focus on clinically relevant heterogeneity and translational challenges. Against this background, the following section discusses the clinical performance of FAPI radiopharmaceuticals across different tumour entities, ligand designs, and radionuclide platforms.

Radiopharmaceuticals based on labelled [^68^Ga]gallium/[^18^F]fluoride/[^64^Cu]copper -FAPI have shown promise as novel diagnostic tools for at least 30 types of cancers associated with FAP and CAFs, particularly in tumours where fibroblasts constitute up to 90% of the tumour mass (especially: pancreatic, colorectal, breast, lung, head and neck, gastric, liver primary cancers and lymph node metastases). Different forms of FAPI have various applications. For example, monomeric forms may be beneficial for rapid imaging (e.g., FAPI-04), but often exhibit lower retention in the target tissue. In contrast, dimers are more metabolically stable and exhibit longer retention in cancer tissue, albeit with a lower background signal in healthy organs, thereby demonstrating better theranostic properties. These observations are consistent with earlier reviews [25,68], which reported the broad applicability of FAPI radiopharmaceuticals in FAP- and CAF-rich malignancies and highlighted structural differences between monomeric and dimeric ligands as key determinants of tumour retention and theranostic performance.

In addition to ligand structure, the choice of radionuclide and production pathway plays a critical role in determining the clinical applicability of FAPI-based imaging. The ease of obtaining the [^68^Ga]gallium isotope from registered generators, the straightforward radiolabelling process, and the high efficiency of the procedure make it a competitive option compared to the complex synthesis of cyclotron-based radiopharmaceuticals. The exceptionally high uptake of [^68^Ga]Ga-FAPI enhances its utility in diagnosing various cancer types, especially in cases where traditional [^18^F]FDG PET/CT faces limitations. Furthermore, [^68^Ga]Ga-DOTA-FAPI-04 PET may complement [^18^F]FDG PET/CT in detecting small malignant lesions. The versatility of [^68^Ga]Ga-FAPI also enables theranostic applications, providing targeted therapy options for cancers associated with fibroblast activation, which are typically immunosuppressive and chemoresistant, making them attractive targets for combination therapies. Notably, [^68^Ga]Ga-FAPI radiopharmaceuticals contain the universal DOTA chelator in their structure, enabling them to be labelled with therapeutic radiopharmaceuticals such as [^177^Lu]lutetium for targeted tumour destruction. Integrating FAPI-targeted radionuclide therapy with chemotherapy, immunotherapy, or other precision medicine approaches may enhance therapeutic efficacy, particularly in tumours that are resistant to treatment, and should be considered.

On the other hand, the ^18^F-labelled FAPI derivatives, such as [^18^F]F-FAPI-42 and [^18^F]F-FAPI-74, due to their extended half-life (109.7 min) and compatibility with cyclotron production of [^18^F]fluoride, enhance scalability and accessibility in clinical settings, offering advantages over shorter-lived [^68^Ga] gallium-based radiotracers.

Despite these promising diagnostics and theranostic applications, several limitations should be noted regarding the use of radiolabelled FAPI. Tumours can vary significantly in their fibroblast content within the TME. Cancers of the breast, prostate, and pancreas contain substantial amounts of activated fibroblasts, while tumours in the brain, kidney, and ovary typically have lower levels of these cells. Additionally, inflammation-induced fibrosis can lead to false-positive uptake of [^68^Ga]Ga-DOTA-FAPI-04, a significant limitation to consider. Careful interpretation of [^68^Ga]Ga-DOTA-FAPI-04 PET/CT images is necessary, particularly for inflammation-related tumours. In this context, quantitative metrics such as SUV values should be interpreted with caution, as overlapping uptake levels may be observed between malignant lesions and benign fibroblast activation. Currently, no validated SUV thresholds exist that reliably differentiate tumour-related FAPI uptake from inflammation- or fibrosis-associated uptake. Therefore, a comprehensive interpretation integrating uptake patterns, anatomical correlates on CT or MRI, clinical history, and, when available, comparative imaging with [^18^F]FDG PET/CT is recommended to improve diagnostic accuracy and avoid misinterpretation.

The diagnostics and therapy involving FAPI-labelled radiopharmaceuticals are associated with numerous unresolved issues requiring further preclinical and clinical research. Several key challenges remain to be addressed before widespread clinical implementation can be achieved:

- Optimising FAPI structure, particularly studying novel multimeric forms to enhance avidity and selectivity while minimising background interference;
- Determining dose thresholds, especially in the context of targeted therapy, and evaluating the long-term safety and efficacy of these doses;
- Investigating the causes of heterogeneous FAP expression in tumours and its impact on the effectiveness of FAPI radiopharmaceuticals;
- Analysing the effects of high FAPI doses on healthy tissues during repeated therapeutic cycles;
- Testing FAPI in combination with chemotherapy, immunotherapy, or signalling pathway inhibitors to evaluate synergy in treating resistant cancers.

These studies are critical to advancing the clinical utility of FAPI-based agents in precision oncology.

This review has several limitations that should be acknowledged. First, although a structured literature search was performed, the present analysis was not conducted as a formal systematic review and may therefore be subject to selection bias. Second, the available evidence on FAPI radiopharmaceuticals is heterogeneous, with many studies characterised by small patient cohorts, retrospective designs, and variability in imaging protocols, radiotracers, and outcome measures, which limits direct comparability. Third, quantitative parameters such as SUV values were reported as presented in the original studies and should be interpreted with caution due to methodological differences. Finally, data on therapeutic applications remain preliminary, and longer follow-up and prospective trials are needed to better define the clinical role of FAPI-based theranostics.

## Figures and Tables

**Table 1:** Comparative pharmacokinetic characteristics of selected FAPI-based radiopharmaceuticals relevant for theranostic applications.

Ligand	Structural Features	Tumour Retention	Clearance Pathway	Lipophilicity	Main Clinical Implication
FAPI-04 [24,35,36]	Monomeric quinoline-based FAP inhibitor.	Rapid tumour uptake with relatively fast washout. High tumour-to-background contrast.	Predominantly renal	Low	Excellent diagnostic imaging, limited therapeutic retention
FAPI-46 [3,30,34]	Modified monomer with increased plasma stability.	Improved tumour retention compared to FAPI-04. Higher tumour uptake and lower uptake in normal organs compared to FAPI-04.	Renal with slower clearance	Moderate	Balanced diagnostic performance with improved theranostic potential
FAPI-2286 [33,37]	Peptide-based FAP-targeting ligand	Prolonged tumour retention.	Renal and hepatobiliary.	Higher	Optimised for radionuclide therapy and theranostic applications

**Table 2:** A comparison of [^68^Ga]FAPI vs. [^18^F]FDG uptake in certain types of cancer based on selected articles.

Site	Radiopharmaceutical	[^68^Ga]FAPI Tumour Uptake (SUV-Based Metrics)	[^18^F]FDG Tumour Uptake (SUV-Based Metrics)	No. of Patients	Age/Sex	Ref.
**Gastrointestinal and Hepatobiliary Tumours**						
Colorectal and pancreatic cancer (P)	[^68^Ga]Ga-DOTA-FAPI-02	7.3	7.4	1	55/M and 31/M	[38]
Gastric cancer (P)	[^68^Ga]Ga-FAPI-04	9.7	4.6	44	61; 3 M/34 F	[43]
Pancreatic cancer (P)	[^68^Ga]Ga-FAPI-04	10.4	5.1	18		[43]
Hepatocellular carcinoma (P)	[^68^Ga]Ga-FAPI-04	5.9 ± 3.4	6.9 ± 5.0	35	59.4 ± 6.9; 24 M/1 F	[49]
Liver cancer	[^68^Ga]Ga-FAPI-04	12.3	3.5	6	-	[51]
**Thoracic Malignancies**						
Lung cancer (P)	[^68^Ga]Ga-FAPI-04	11.9	4.5	8	61.5; 47 M/28 F	[40]
Lung cancer (P)	[^68^Ga]Ga-FAPI-04	9.7	9.8	123	56.1 ± 11.9; 69 M/54 F	[45]
**Head and Neck Tumours**						
Nasopharyngeal carcinoma (P)	[^68^Ga]Ga-FAPI-04	15.8	8.9	6	61.5; 47 M/28 F	[40]
Laryngeal cancer (P)	[^68^Ga]Ga-FAPI-04	14.2	6.6	123	56.1 ± 11.9; 69 M/54 F	[45]
**Breast and Gynaecological Tumours**						
Breast cancer (P)	[^68^Ga]Ga-DOTA.SA.FAPI	6.5 ± 3.3	6.2 ± 1.6	20	46; 20 F	[23]
Ovarian cancer (P)	[^68^Ga]Ga-DOTA-FAPI-04	8.9 ± 0.8	6.7 ± 1.5	2	49; 2 F	[23]
Cervical cancer (P)	[^68^Ga]Ga-FAPI-04	11.3	8.2	3	61.5; 47 M/28 F	[40]
**Neuroendocrine and CNS Tumours**						
Neuroendocrine tumours (P)	[^68^Ga]Ga-FAPI-04	7.8	5.7	3	61.5; 47 M/28 F	[40]
Glioma (P)	[^68^Ga]Ga-FAPI-04	3.6	5.9	4	61.5; 47 M/28 F	[40]
Intracranial tumours (P)	[^68^Ga]Ga-FAPI-04	5.2	17.4	123	56.1 ± 11.9; 69 M/54 F	[45]
**Genitourinary Tumours**						
Renal cell carcinoma (P)	[^68^Ga]Ga-FAPI-04	7.3	6.3	6	62; 7 F/13 M	[50]
Prostate cancer (P)	[^68^Ga]Ga-FAPI-04	3.4	7.5	123	56.1 ± 11.9; 69 M/54 F	[45]
**Metastatic Lesions (All Sites)**						
Lymph node metastases (M)	[^68^Ga]Ga-DOTA-FAPI-02 *n =* 6 [^68^Ga]Ga-DOTA-FAPI-04 *n =* 32 [^68^Ga]Ga-DOTA-FAPI-46 *n =* 32 [^68^Ga]Ga-DOTA-FAPI-74 *n =* 1	7.9	11.2	71 patients (164 metastatic lesions were found in 43 patients)	60/-	[35]
Liver metastases (M)		9.8	8.8			
Bone metastases (M)		7.8	7.5			
Lung metastases (M)		6.7	11.5			
Other M (plural, peritoneal, soft tissue M)		10.7	8.2			

**Notes:** Abbreviations: P, primary tumour; M, metastatic lesion; in column 6, F = female, M = male, *n*–the number of patients who were examined using a given radiopharmaceutical. Quantitative uptake values are reported as provided in the original publications and include average SUVmax and, where specified, mean SULpeak.

## Data Availability

No new data were created or analysed in this study. Data sharing is not applicable to this article.
